# Clinical and Radiological Changes of Ankle in Knee Osteoarthritis With Varus After Total Knee Arthroplasty: A Systematic Review

**DOI:** 10.3389/fsurg.2021.713055

**Published:** 2021-08-30

**Authors:** Zhiwei Feng, Ming Ma, Yaobin Wang, Chenfei Yang, Zhongcheng Liu, Yayi Xia

**Affiliations:** ^1^Department of Orthopaedics, Lanzhou University Second Hospital, Lanzhou, China; ^2^Department of Orthopaedics, Nanchong Central Hospital, The Second Clinical Institute of North Sichuan Medical College, Nanchong, China; ^3^North Sichuan Medical College, Nanchong, China

**Keywords:** systematic review, ankle, knee osteoarthritis, varus knee, total knee arthroplasty

## Abstract

**Background:** Arthritis with severe varus deformity remains a challenge in total knee arthroplasty (TKA). Until recently, surgeons aimed at a neutral lower limb alignment when performing a TKA. However, the impact of TKA on the ankle joint has been ignored. Therefore, we conducted a systematic review to assess the clinical and radiological changes in the ankle joint after TKA on knees with severe varus deformity.

**Methods:** A systematic search was conducted in four English (PubMed, Embase, Cochrane Library, and Web of Science) and four Chinese (CBM, VIP, CNKI, and Wan Fang Database) databases. Screening of literature and extraction of data were independently performed by two researchers. The modified methodological index for non-randomized studies (MINORS) was used to assess the quality.

**Results:** A total of eight studies were eligible, namely, four prospective and four retrospective studies. TKA resulted in a negative clinical effect in the ankle joint in patients with ankle osteoarthritis. Seven studies reported changes in the mechanical tibiofemoral angle, and four studies reported radiological changes in the hindfoot. The mean score of the MINORS was 9.8 out of eight (9–11).

**Conclusion:** As a result of the correction of the knee osteoarthritis with severe varus deformity following mechanically aligned TKA, the radiological malalignment of the ankle joint was improved. However, some patients experience increased ankle pain after undergoing TKA, especially, if there was a residual knee varus deformity, a stiff hindfoot with varus deformity, or ankle arthritis.

## Introduction

Concomitant ankle osteoarthritis (OA) is frequently found in patients who undergo TKA, which is a classic surgery for elderly patients, who have experienced knee osteoarthritis (KOA), with multiple joint degenerative changes (1–3). Furthermore, malalignment of the knee joint caused by KOA could induce ankle tilt that would further aggravate ankle OA (4). Nonetheless, a few patients experienced increased or newly developed ankle pain after TKA (5). Surgeons lack a comprehensive consideration of the ankle condition of the patient when performing TKA.

Most of the patients with knee arthritis who undergo TKA have varying degrees of knee joint deformity, which may be associated with hindfoot deformity (6–8). Previous studies reported an association between varus alignment of the knee joint and valgus alignment of the hindfoot in patients with KOA (7, 8). Because the mild deformity of the knee joint can be compensated by the subtalar joint (8), the knee joint alignment modification after TKA can affect the hindfoot alignment. Previous studies have demonstrated that improvements in the hindfoot alignment have been observed in patients who experience OA after TKA (7, 9, 10). However, hindfoot valgus may show little improvement and persist after TKA (7, 9).

Patients with a hindfoot deformity present a particular challenge when undergoing TKA. Clarifying the relationship between hindfoot alignment and TKA is useful to change the current state. Thus, this systematic review attempted to assess prospective and retrospective cohort studies to determine the relationship between the radiological changes and clinical symptoms of the ankle joint following acute correction of the lower alignment using TKA for severe varus knee.

## Methods

### Literature Search

A systematic search was conducted in four English (PubMed, Embase, Cochrane Library, and Web of Science) and four Chinese (CBM, VIP, CNKI, and Wan Fang Database) databases from inception to December 2020 for relevant non-randomized controlled trials (nRCTs). The search was based on the following search terms: (“Arthroplasty, Replacement, Knee” OR “Total knee arthroplasty” OR “Knee replacement” OR “TKA”) AND (“varus knee”) AND (“ankle alignment” OR “ankle deformity” OR “hindfoot alignment” OR “hindfoot deformity”). Additional records were obtained by screening the references. Detailed inclusion and exclusion criteria were formulated to review the results of the search by two reviewers (Feng ZH W and Ma M); the abstract was screened first before reading the full text.

### Literature Inclusion and Exclusion

The inclusion criteria were as follows: (1) nRCT, prospective or retrospective studies, and case control studies; (2) patients who underwent primary TKA as research participants; (3) with mechanical tibiofemoral angle (MTA), hindfoot alignment angle (HA), American Orthopedic Foot and Ankle Score (AOFAS), talar tilt (Tt), medial ankle joint space (MAJS), and medial ankle clear space (MACS) as indicators of the postoperative results; and (4) all retained studies should have the same hip knee ankle (HKA) goal, i.e., neutral mechanical alignment (MA). If not, the study should be excluded. The exclusion criteria were as follows: (1) diagnosis of a disease other than primary KOA; (2) KOA with valgus malalignment or rheumatoid arthritis; (3) history of femoral or tibial fractures in the past; (4) incomplete or missing research data; and (5) research data from animal experiments or theoretical analysis. Repeated publications, letters, case reports, comments, conference abstracts, or books were also excluded.

### Data Extraction

We performed data extraction of all the included documents with predesigned tables, and all the studies were independently retrieved and assessed by two reviewers (ZH W Feng and M Ma). The extracted data should include the following: (1) title, first author, publication time, study design, clinical indicators, and radiological indicators; (2) patient characteristics such as number of patients, age, sex, number of patients who underwent TKA, and follow-up time; and (3) study details such as clinical results, radiological results, and conclusions. The extracted information was cross-checked to ensure accuracy. When there were disagreements, the decision was made by a third person.

### Quality Assessment

The methodological quality of nRCTs was assessed using the modified MINORS (11). Eight questions for the nRCTs were used to score the relevant aspects of each study: a clear research purpose (I), continuous follow-up of patients (II), prospective research (III), suitable research purpose (IV), unbiased assessment of the research outcome (V), an appropriate follow-up period for the study (VI), the lost-to-follow-up rate was < 5% (VII), and perform a prospective calculation of the sample size (VIII).

The score criteria are as follows: not reported scored as 0, reported but inadequate scored as 1, and reported and adequate scored as 2. The MINORS score was assessed separately by the two reviewers (ZH W Feng and M Ma). Then, the categories were determined according to the study by Ekhtiari et al. (12): “0–4 points” were categorized as very low; “5–8 points” as low; “9–12 points” as good; and “13–16 points” as excellent.

## Results

### Study Selection

**Figure 1:**
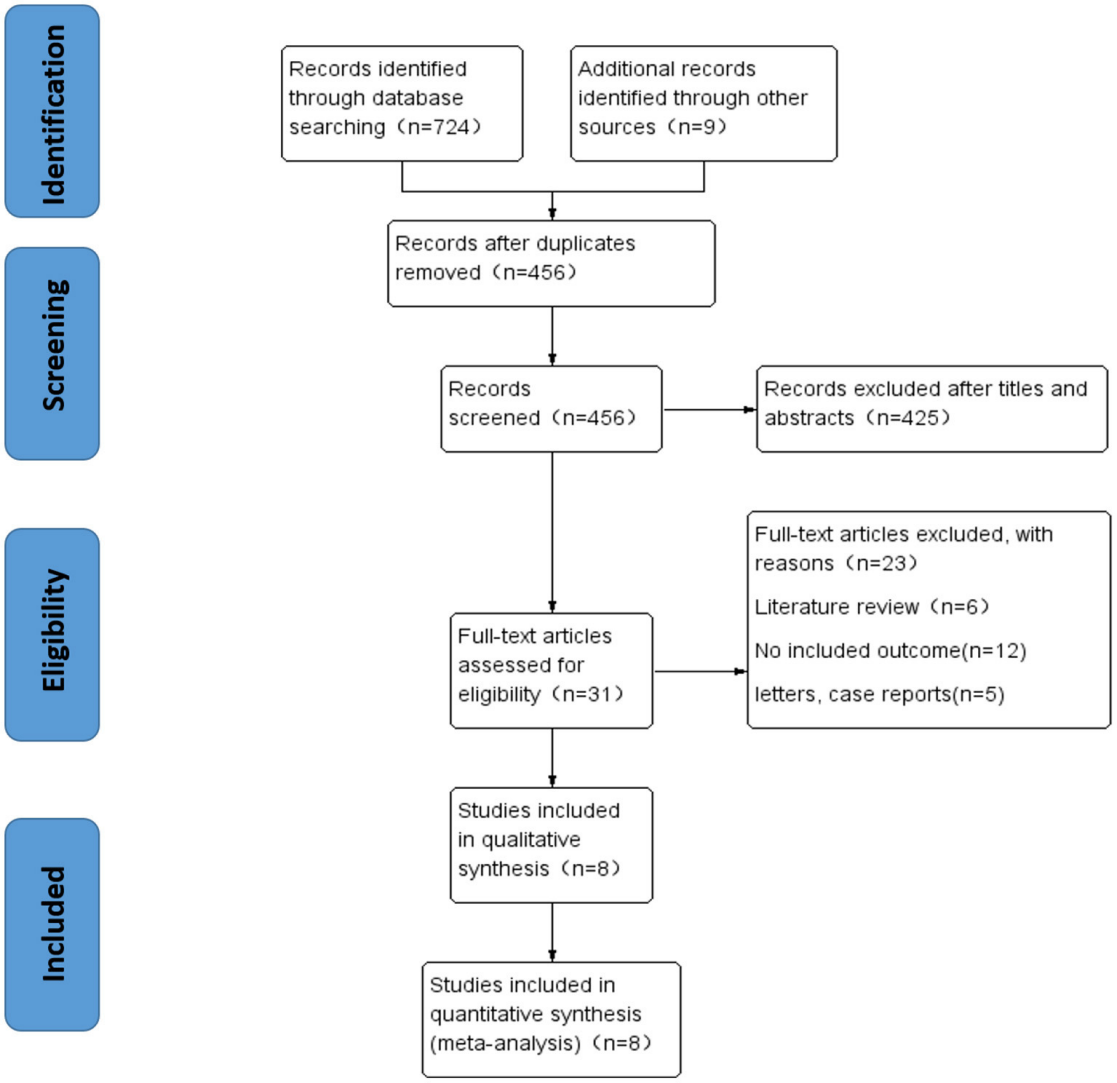
Flowchart for the identification of eligible studies.

A total of 733 studies were included in this study. After removing 277 duplicates, the titles, abstracts, and full articles were screened, and 425 studies were further excluded. A total of eight eligible articles were considered for further analysis. The results of the study selection process are presented in Figure 1.

### Study Characteristics

A total of 913 patients and 1,157 knees were included in the eligible studies. All included studies were observational studies, and their publication years ranged from 2012 to 2019. The follow-up duration was extended from 6 months to 3 years. Tables 1, 2 show the characteristics of the included study.

**Table 1:** Study details.

**References**	**Country**	**Design**	**Cohort description**	**Total number (people/knee)**	**Mean age (year)**	**Male (M/F)**	**Follow-up**	**Outcomes**
Cho et al. (13)	South Korea	Pc	Varus <10° KOA, Varus ≥ 10° KOA	117/195	69.1 ± 6.1	N/R	2 years	①②
Okamoto et al. (5)	Japan	Rc	Varus ≤ 6° KOA, Varus > 6° KOA	75/80	72.5 (range, 58–85)	8/67	2 years	①③④
Chang et al. (14)	South Korea	Rc	Varus KOA + Ankle OA, Varus KOA + NO Ankle OA	56/99	70 ± 6.8	6/50	2 years	①②④⑤⑥
Gursu et al. (15)	Turkey	Rc	Varus > 10° KOA	78/80	67 (range, 54–78)	18/60	3 years	①④⑥
Jeong et al. (16)	South Korea	Pc	Varus KOA	331/375	68.3 ± 7.8	23/308	6 months	①②④
Palanisami et al. (17)	India	Pc	Varus > 10° KOA	91/121	63.4 ± 7.6	29/62	1 year	①②③
Lee et al. (18)	South Korea	Rc	Varus + valgus KOA	110/142	65.8	N/R	3 years	④⑤⑥
Kim et al. (19)	South Korea	Pc	Varus KOA	55/ 65	N/R	N/R	2 years	①②③④⑤⑥

*PC, prospective cohort; RC, retrospective cohort; N/R, not reported; ①, MTA or the angle formed between the mechanical axis of the femur and the mechanical axis of the tibia; ②, HA or the angle between the diaphyseal axis of the tibia and the longitudinal axis of the calcaneus; ③, AOFAS or the American Orthopedic Foot and Ankle Score; ④, Tt or the angle between the distal tibial surface and the upper talus; ⑤, MAJS or the distance between the medial of the talar dome and the distal tibial surface; ⑥, MACS or the distance between the medial articular surface of the talus and the medial malleolus articular surface*.

**Table 2:** Study details.

**References**	**Clinical indicator**		**Radiographic indicator**		**Correlation**	**Conclusion**
	**Knee**	**Ankle**	**Knee**	**Ankle**		
Cho et al. (13)	N/R	N/R	Significant difference of MTA after TKA (*p* < 0.001)	Significant difference of HA after TKA (*p* < 0.001)	Correlation between MTA and HA before and after TKA (*p* < 0.05).	The hindfoot valgus deformity in patients with knee varus deformity does not require to be corrected before TKA.
Okamoto et al. (5)	Significant difference of KSS, KSFS after TKA (*p* < 0.05)	Significant difference of AOFAS after TKA (*p* < 0.05)	Significant difference of MTA after TKA (*p* < 0.001)	Significant difference of Tt after TKA (*p* < 0.05)	Correlation between MTA and Tt before and after TKA (*p* < 0.05).	Patients with severe knee deformity would experience persistent hindfoot pain and valgus alignment after TKA.
Chang et al. (14)	N/R	Patients with ankle OA had increased ankle pain and worse clinical outcomes after TKA (*p* < 0.05).	Significant difference of MTA after TKA (*p* < 0.001)	Significant difference t of Tt, MAJS, and MACS after TKA (*p* < 0.05)	Correlation between MTA and Tt, MAJS, and MACS before and after TKA (*p* < 0.05).	The ankle OA is related to the increased ankle pain after TKA and adversely affects the clinical outcomes.
Gursu et al. (15)	N/R	N/R	Significant difference of MTA after TKA (*p* < 0.001)	Significant difference of Tt and MACS after TKA (*p* < 0.05)	N/R	The correction of severe knee varus deformity following TKA would lead to ankle malalignment.
Jeong et al. (16)	N/R	N/R	Significant difference of MTA after TKA (*p* < 0.001)	Significant difference t of HA and Tt after TKA (*p* < 0.001)	Correlation between MTA and HA, Tt before and after TKA (*p* < 0.05).	The correction of knee varus deformity after TKA would lead to compensatory changes, which occurred at the ankle and the subtalar joints.
Palanisami et al. (17)	Significant difference of OKS after TKA (*p* < 0.001)	Significant difference of AOFAS after TKA (*p* < 0.001)	Significant difference of MTA after TKA (*p* < 0.001)	Significant difference of HA after TKA (*p* < 0.001)	Correlation between MTA and HA before and after TKA (*p* < 0.05).	The knee and hindfoot alignment in patients with knee varus deformity can be restored by TKA.
Lee et al. (18)	N/R	N/R	Significant difference of MTA after TKA (*p* < 0.001)	Significant difference of Tt, MAJS, and MACS after TKA (*p* < 0.05)	Correlation between MTA and Tt, MAJS, and MACS before and after TKA (*p* < 0.05).	The correction of knee varus deformity after TKA would lead to radiographically progressed ankle arthritis.
Kim et al. (19)	N/R	Significant difference of AOFAS and VAS after TKA (*p* < 0.01)	Significant difference of MTA after TKA (*p* < 0.001)	Significant difference of Tt, HA after TKA (*p* < 0.05)	Correlation between MTA and Tt, HA before and after TKA (*p* < 0.05).	The correction of knee varus deformity after TKA would lead to more serious ankle pain.

*N/R, not reported; KSS, Knee Society Score; KSFS, Knee Society Functional Score; VAS, visual analog scale; OKS, Oxford Knee Score; MTA, mechanical tibiofemoral angle; HA, hindfoot alignment angle; AOFAS, American Orthopedic Foot and Ankle Score; Tt, talar tilt; MAJS, medial ankle joint space; MACS, medial ankle clear space*.

### Quality Assessment

We used the MINORS criteria to assess the methodological quality of the nRCTs. The categories were determined according to the research of Ekhtiari et al. (12). The quality evaluation of the included research literature is shown in Table 3.

**Table 3:** MINORS score.

**References**	**I**	**II**	**III**	**IV**	**V**	**VI**	**VII**	**VIII**	**Total score**
Cho et al. (13)	2	1	1	2	1	2	1	1	11
Okamoto et al. (5)	2	1	0	2	1	2	1	1	10
Chang et al. (14)	2	1	0	2	1	2	0	1	9
Gursu et al. (15)	2	1	0	2	1	2	0	1	9
Jeong et al. (16)	2	1	1	2	1	1	0	1	9
Palanisami et al. (17)	2	1	1	2	1	2	1	1	11
Lee et al. (18)	2	1	0	2	1	2	0	1	9
Kim et al. (19)	2	1	1	2	1	2	1	1	11

*I–VIII, question; MINORS score, not reported scored as 0, reported but inadequate scored as 1, reported and adequate scored as 2*.

### Research Details

After removing duplicate studies and applying the inclusion and exclusion criteria, eight studies were eligible for further analysis (Figure 1). The eligible studies included a total of 913 patients and 1,157 knees, which included four prospective cohort studies (13, 16, 17, 19) and four retrospective studies (5, 14, 15, 18). Seven studies (5, 13–17, 19) only studied the varus KOA, and one study (18) investigated both varus and valgus KOA. The characteristics of the eligible studies were extracted by two researchers (Table 1).

All the clinical and radiological outcomes of the knee and hindfoot after TKA are shown in Table 2. Four studies (5, 14, 17, 19) have reported the specific clinical outcomes of the knee and hindfoot after TKA. Okamoto et al. (5) reported that patients with hindfoot deformity after TKA would have a significant improvement in Knee Society Score, Knee Society Functional Score, and AOFAS. Chang et al. (14) reported that patients with ankle OA experienced increased ankle pain and poor clinical prognosis after TKA. Meanwhile, Palanisami et al. (17) reported that the Oxford Knee Score and AOFAS of patients with foot deformities after TKA significantly improved. Kim et al. (19) also reported that a persistent ankle varus deformity could be attributed to increased ankle pain after TKA.

Seven studies (5, 13–17, 19) compared the change in MTA pre- and postoperative, which found significant postoperative improvement of MTA after TKA. Five studies (13, 14, 16, 17, 19) included a radiological analysis of HA before and after TKA, and found significant improvement in hindfoot deformity. Cho et al. (13) also found that there was a weak negative correlation between the preoperative HA and MTA (−0.484, *p* < 0.001), and a very weak correlation between the postoperative MTA and postoperative HA at 6 weeks (−0.147, *p* = 0.040). Six studies (5, 14–16, 18, 19) included a radiological analysis of pre- and postoperative Tt, which found significant improvement. Lee et al. (18) found that the incidence of ankle arthritis would obviously increase when the preoperative Tt was closer to the ankle medial or when the angle of correction was greater after TKA. Lastly, three studies (14, 18, 19) included a radiological analysis of MAJS and MACS pre- and postoperatively, which showed a significant difference.

## Discussion

Traditionally, obtaining a neutral lower limb alignment after a TKA was perceived as the ideal goal (20–23). However, since very few individuals have such anatomy, it implies a significant modification for most. Freeman et al. (24) first introduced the concept of right-angled femoral and tibial bone cuts in TKA-MA. Subsequently, Scuderi et al. (25), raised the importance of balancing the resulting medial–lateral and flexion–extension joint gaps. MA technique *gradually* became the gold standard in TKA. However, the recent studies found that MA-TKA generates disappointing efficacies (26, 27), probably due to MA-TKA produces a non-physiological prosthetic knee by changing the native anatomy, physiological ligament balance, and kinematics (28–31). Stephen Howell developed the kinematic alignment (KA) technique (28, 30). KA-TKA aims to generate a more physiological prosthetic knee joint by restoring the inherent knee joint anatomy and physiological soft tissue balance of the individual. Several studies have suggested that KA-TKA can accurately locate the position of the prosthesis (32), and also restore the native anatomy of the knee (32, 33) and physiological laxity (34, 35). The ultimate goal of TKA is to restore the anatomy and kinematics of native knees and provide a forgotten joint.

Recent studies have found that the alignment of the ankle joint and hindfoot could be affected by the malalignment of the lower limb (4, 5, 8, 36, 37). Moreover, the hindfoot deformity is closely associated with the lower limb alignment (38), and the clinical symptoms and radiographic outcome changes of the ankle joint and hindfoot in patients who underwent TKA would be influenced by changes in lower limb alignment (5). Chang et al. (14) found that there was less radiographic alignment change in the ankle and hindfoot when patients had ankle OA and increased ankle pain after TKA; and patients with a stiff hindfoot who underwent TKA experienced increased ankle pain, probably because of the inability to reorient the ankle after TKA. Nevertheless, different findings have been reported by these studies, but there have been no comprehensive reviews of this issue in the literature so far.

In this systematic review, we found that TKA may affect the clinical and radiological outcomes of patients with hindfoot deformity before TKA. In addition, the HA before TKA had a weak negative correlation with MTA (13). Clinically, four studies (5, 14, 17, 19) detected an obvious improvement in AOFAS at the hindfoot after TKA. However, increased ankle pain after TKA has also been reported in the patients with ankle OA or persistent knee deformity (14, 19). When patients have a residual deformity and pain of the hindfoot 6 weeks after TKA, they must receive active treatment because further improvement cannot be expected (13). Furthermore, five studies (13, 14, 16, 17, 19) reported an obvious radiological improvement in hindfoot alignment after TKA. Kim et al. (19) also reported a relationship between residual varus deformity and ankle pain after TKA.

**Figure 2:**
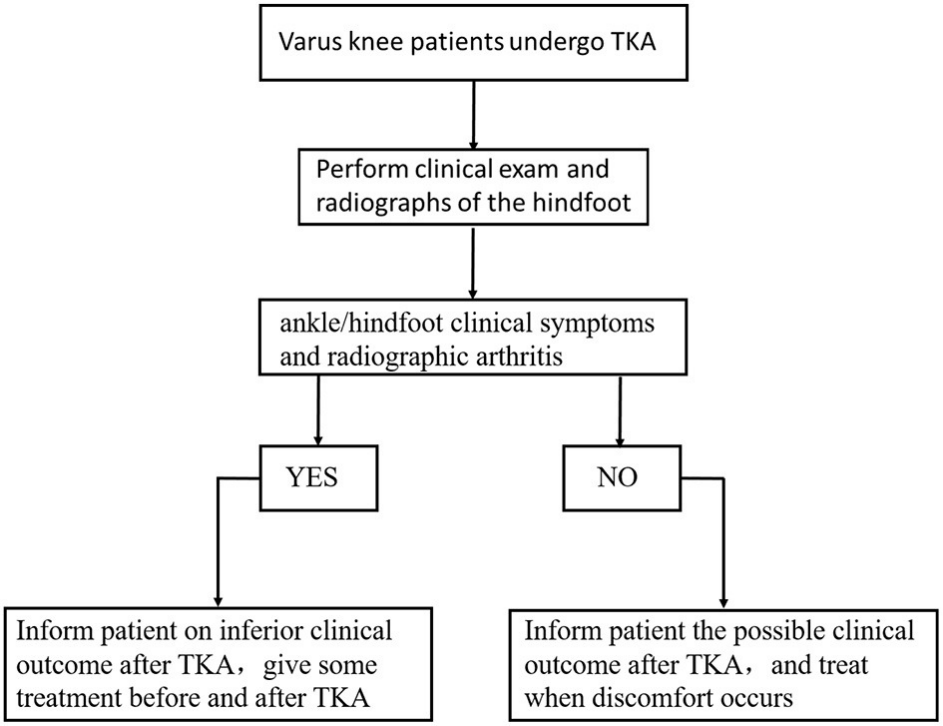
Flowchart in patients undergoing TKA.

Figure 2 shows the flowchart for the general treatment of patients with knee varus and ankle stiffness before and after TKA. Before TKA, the clinicians should perform clinical and radiological examinations of the ankle/hindfoot. If the patient has clinical or imaging problems with the ankle/hindfoot, the clinicians should inform the patient about the results and provide some treatment before and after TKA. If the patient does not have ankle/hindfoot problems before TKA, the clinician should tell them about possible problems after TKA. Patients should be informed and treated when discomfort occurs.

This study has several limitations. First, eligible papers according to the search criteria used were not identified completely. Second, the eligible studies found for this systematic review had a relatively small number and a relatively high heterogeneous group, which could not be studied by meta-analysis. Third, the follow-up time in two studies (16, 17) was <2 years, which limited the ability to draw a long-term conclusion.

In conclusion, an improvement in the clinical function and radiological alignment of the hindfoot can be achieved following TKA. However, when patients had concomitant ankle OA with hindfoot stiffness, there is an increased ankle pain and a worse clinical outcome after TKA.

Therefore, the comprehensive preoperative evaluation of surgeons of the ankles of patients who complain of pain pre- and postoperatively and the correction of alignment during TKA should be given careful attention.

## Data Availability Statement

The raw data supporting the conclusions of this article will be made available by the authors, without undue reservation.

## Author Contributions

ZF and MM worked on literature search, study review, and manuscript draft. YW, CY, and ZL prepared the tables and figures. YX worked on manuscript review, process supervision, and draft revision. All authors have read and approved the final manuscript.

## Conflict of Interest

The authors declare that the research was conducted in the absence of any commercial or financial relationships that could be construed as a potential conflict of interest.

## Publisher's Note

All claims expressed in this article are solely those of the authors and do not necessarily represent those of their affiliated organizations, or those of the publisher, the editors and the reviewers. Any product that may be evaluated in this article, or claim that may be made by its manufacturer, is not guaranteed or endorsed by the publisher.

## References

[1] Ranawat CS Park CN White PB Meftah M Bogner EA Ranawat AS. Severe hand osteoarthritis strongly correlates with major joint involvement and surgical intervention. J Arthroplasty. (2016) 31:1693–7. 10.1016/j.arth.2016.01.044 26968694

[2] Tallroth K Harilainen A Kerttula L Sayed R. Ankle osteoarthritis is associated with knee osteoarthritis. Conclusions based on mechanical axis radiographs. Archiv Orthopaed Trauma Surg. (2008) 128:555–60. 10.1007/s00402-007-0502-9 18030482

[3] Cho HJ Morey V Kang JY Kim KW Kim TK. Prevalence and risk factors of spine, shoulder, hand, hip, and knee osteoarthritis in community-dwelling Koreans Older Than Age 65 years. Clin Orthopaed Relat Res. (2015) 473:3307–14. 10.1007/s11999-015-4450-3 26162413 PMC4562942

[4] Gao F Ma J Sun W Guo W Li Z Wang W. The influence of knee malalignment on the ankle alignment in varus and valgus gonarthrosis based on radiographic measurement. Eur J Radiol. (2016) 85:228–32. 10.1016/j.ejrad.2015.11.021 26724670

[5] Okamoto Y Otsuki S Jotoku T Nakajima M Neo M. Clinical usefulness of hindfoot assessment for total knee arthroplasty: persistent post-operative hindfoot pain and alignment in pre-existing severe knee deformity. Knee Surg Sports Traumatol Arthrosc. (2017) 25:2632–9. 10.1007/s00167-016-4122-1 27056693

[6] Desai SS Shetty GM Song H-R Lee SH Kim TY Hur CY. Effect of foot deformity on conventional mechanical axis deviation and ground mechanical axis deviation during single leg stance and two leg stance in genu varum. Knee. (2007) 14:452–7. 10.1016/j.knee.2007.07.009 17825567

[7] Mullaji A Shetty GM. Persistent hindfoot valgus causes lateral deviation of weightbearing axis after total knee arthroplasty. Clin Orthopaed Relat Res. (2011) 469:1154–60. 10.1007/s11999-010-1703-z 21120711 PMC3048272

[8] Norton AA Callaghan JJ Amendola A Phisitkul P Wongsak S Liu SS. Correlation of knee and hindfoot deformities in advanced knee OA: compensatory hindfoot alignment and where it occurs. Clin Orthopaed Relat Res. (2015) 473:166–74. 10.1007/s11999-014-3801-9 25024033 PMC4390938

[9] Chandler JT Moskal JT. Evaluation of knee and hindfoot alignment before and after total knee arthroplasty: a prospective analysis. J Arthroplasty. (2004) 19:211–6. 10.1016/j.arth.2003.09.007 14973865

[10] Hara Y Ikoma K Arai Y Ohashi S Maki M Kubo T. Alteration of hindfoot alignment after total knee arthroplasty using a novel hindfoot alignment view. J Arthroplasty. (2015) 30:126–9. 10.1016/j.arth.2014.07.026 25155238

[11] Slim K Nini E Forestier D Kwiatkowski F Panis Y Chipponi J. Methodological index for non-randomized studies (minors): development and validation of a new instrument. ANZ J Surg. (2003) 73:712–6. 10.1046/j.1445-2197.2003.02748.x 12956787

[12] Ekhtiari S Horner NS Bedi A Ayeni OR Khan M. The learning curve for the latarjet procedure: a systematic review. Orthop J Sports Med. (2018) 6:2325967118786930. 10.1177/2325967118786930 30090836 PMC6077900

[13] Cho WS Cho HS Byun SE. Changes in hindfoot alignment after total knee arthroplasty in knee osteoarthritic patients with varus deformity. Knee Surg Sports Traumatol Arthrosc. (2017) 25:3596–604. 10.1007/s00167-016-4278-8 27527338

[14] Chang CB Jeong JH Chang MJ Yoon C Song MK Kang SB. Concomitant ankle osteoarthritis is related to increased ankle pain and a worse clinical outcome following total knee arthroplasty. J Bone Joint Surg Am. (2018) 100:735–41. 10.2106/JBJS.17.00883 29715221

[15] Gursu S Sofu H Verdonk P Sahin V. Effects of total knee arthroplasty on ankle alignment in patients with varus gonarthrosis: do we sacrifice ankle to the knee? Knee Surg Sports Traumatol Arthrosc. (2016) 24:2470–5. 10.1007/s00167-015-3883-2 26590564

[16] Jeong BO Kim TY Baek JH Jung H Song SH. Following the correction of varus deformity of the knee through total knee arthroplasty, significant compensatory changes occur not only at the ankle and subtalar joint, but also at the foot. Knee Surg Sports Traumatol Arthrosc. (2018) 26:3230–7. 10.1007/s00167-018-4840-7 29349665

[17] Palanisami DR Rajasekaran RB Reddy PK Natesan R Sethuraman A Rajasekaran S. Foot loading pattern and hind foot alignment are corrected in varus knees following total knee arthroplasty: a pedobarographic analysis. Knee Surg Sports Traumatol Arthrosc. (2020) 28:1861–7. 10.1007/s00167-019-05629-6 31312876

[18] Lee JH Jeong BO. Radiologic changes of ankle joint after total knee arthroplasty. Foot Ankle Int. (2012) 33:1087–92. 10.3113/FAI.2012.1087 23199858

[19] Kim CW Gwak HC Kim JH Lee CR Kim JG Oh M. Radiologic factors affecting ankle pain before and after total knee arthroplasty for the varus osteoarthritic knee. J Foot Ankle Surg. (2018) 57:865–9. 10.1053/j.jfas.2018.02.002 29779992

[20] Abdel MP Oussedik S Parratte S Lustig S Haddad FS. Coronal alignment in total knee replacement: historical review, contemporary analysis, and future direction. Bone Joint J. (2014) 96-B:857–62. 10.1302/0301-620X.96B7.33946 24986936

[21] Li Z Esposito CI Koch CN Lee Y-Y Padgett DE Wright TM. Polyethylene damage increases with varus implant alignment in posterior-stabilized and constrained condylar knee arthroplasty. Clin Orthopaed Relat Res. (2017) 475:2981–91. 10.1007/s11999-017-5477-4 28822068 PMC5670063

[22] van Hamersveld KT Marang-van de Mheen PJ Nelissen RGHH. The effect of coronal alignment on tibial component migration following total knee arthroplasty: a cohort study with long-term radiostereometric analysis results. J Bone Joint Surg Am. (2019) 101:1203–12. 10.2106/JBJS.18.00691 31274722

[23] Almaawi AM Hutt JRB Masse V Lavigne M Vendittoli PA. The impact of mechanical and restricted kinematic alignment on knee anatomy in total knee arthroplasty. J Arthroplasty. (2017) 32:2133–40. 10.1016/j.arth.2017.02.028 28302462

[24] Freeman MA Swanson SA Todd RC. Total replacement of the knee using the Freeman-Swanson knee prosthesis. 1973. Clin Orthop Relat Res. (2003) 2003:4–21. 10.1097/01.blo.0000093886.12372.74 14646735

[25] Scuderi GR Scott WN Tchejeyan GH. The insall legacy in total knee arthroplasty. Clin Orthop Relat Res. (2001) 2001:3–14. 10.1097/00003086-200111000-00002 11716399

[26] Carr AJ Robertsson O Graves S Price AJ Arden NK Judge A. Knee replacement. Lancet. (2012) 379:1331–40. 10.1016/S0140-6736(11)60752-6 22398175

[27] Collins M Lavigne M Girard J Vendittoli PA. Joint perception after hip or knee replacement surgery. Orthop Traumatol Surg Res. (2012) 98:275–80. 10.1016/j.otsr.2011.08.021 22459101

[28] Rivière C Lazic S Boughton O Wiart Y Vïllet L Cobb J. Current concepts for aligning knee implants: patient-specific or systematic? EFORT Open Rev. (2018) 3:1–6. 10.1302/2058-5241.3.170021 29657839 PMC5890125

[29] Rivière C Iranpour F Auvinet E Aframian A Asare K Harris S. Mechanical alignment technique for TKA: are there intrinsic technical limitations? Orthop Traumatol Surg Res. (2017) 103:1057–67. 10.1016/j.otsr.2017.06.017 28888523

[30] Rivière C Iranpour F Auvinet E Howell S Vendittoli PA Cobb J. Alignment options for total knee arthroplasty: a systematic review. Orthop Traumatol Surg Res. (2017) 103:1047–56. 10.1016/j.otsr.2017.07.010 28864235

[31] Blakeney W Beaulieu Y Puliero B Kiss MO Vendittoli PA. Bone resection for mechanically aligned total knee arthroplasty creates frequent gap modifications and imbalances. Knee Surg Sports Traumatol Arthrosc. (2020) 28:1532–41. 10.1007/s00167-019-05562-8 31201441

[32] Rivière C Iranpour F Harris S Auvinet E Aframian A Chabrand P. The kinematic alignment technique for TKA reliably aligns the femoral component with the cylindrical axis. Orthop Traumatol Surg Res. (2017) 103:1069–73. 10.1016/j.otsr.2017.06.016 28870873

[33] Nedopil AJ Singh AK Howell SM Hull ML. Does calipered kinematically aligned TKA restore native left to right symmetry of the lower limb and improve function? J Arthroplasty. (2018) 33:398–406. 10.1016/j.arth.2017.09.039 29074324

[34] Shelton TJ Howell SM Hull ML. A total knee arthroplasty is stiffer when the intraoperative tibial force is greater than the native knee. J Knee Surg. (2019) 32:1008–14. 10.1055/s-0038-1675421 30414168

[35] Koh IJ Lin CC Patel NA Chalmers CE Maniglio M Han SB. Kinematically aligned total knee arthroplasty reproduces more native rollback and laxity than mechanically aligned total knee arthroplasty: a matched pair cadaveric study. Orthop Traumatol Surg Res. (2019) 105:605–11. 10.1016/j.otsr.2019.03.011 31006644

[36] Lee KM Chang CB Park MS Kang SB Kim TK Chung CY. Changes of knee joint and ankle joint orientations after high tibial osteotomy. Osteoarthritis Cartilage. (2015) 23:232–8. 10.1016/j.joca.2014.11.001 25450843

[37] Gao F Ma J Sun W Guo W Li Z Wang W. Radiographic assessment of knee-ankle alignment after total knee arthroplasty for varus and valgus knee osteoarthritis. Knee. (2017) 24:107–15. 10.1016/j.knee.2016.09.023 27856127

[38] Burssens ABM Buedts K Barg A Vluggen E Demey P Saltzman CL. Is lower-limb alignment associated with hindfoot deformity in the coronal plane? a weightbearing CT analysis. Clin Orthopaed Relat Res. (2020) 478:154–68. 10.1097/CORR.0000000000001067 31809289 PMC7000051

